# Modelling fine-sliced three dimensional electron diffraction data with dynamical Bloch-wave simulations

**DOI:** 10.1107/S2052252522011290

**Published:** 2023-01-01

**Authors:** Anton Cleverley, Richard Beanland

**Affiliations:** a Department of Chemistry, University of Warwick, Coventry CV4 7AL, United Kingdom; b Department of Physics, University of Warwick, Coventry CV4 7AL, United Kingdom; Ben-Gurion University of the Negev, Israel

**Keywords:** 3D electron diffraction, 3D-ED, dynamical refinement, computational modelling, dynamical simulations

## Abstract

Recent interest in structure solution and refinement using electron diffraction (ED) has been fuelled by its inherent advantages when applied to crystals of sub-micrometre size, as well as its better sensitivity to light elements. Currently, data are often processed with software written for X-ray diffraction, using the kinematic theory of diffraction to generate model intensities – despite the inherent differences in diffraction processes in ED. Here, dynamical Bloch-wave simulations are used to model continuous-rotation electron diffraction data, collected with a fine angular resolution (crystal orientations of ∼0.1°). This fine-sliced data allows a re-examination of the corrections applied to ED data. A new method is proposed for optimizing crystal orientation, and the angular range of the incident beam and the varying slew rate are taken into account. Observed integrated intensities are extracted and accurate comparisons are performed with simulations using rocking curves for a (110) lamella of silicon 185 nm thick. *R*_1_ is reduced from 26% with the kinematic model to 6.8% using dynamical simulations.

## Abstract 2

Fine-sliced cRED data allows dynamical electron scattering to be investigated with high precision. Experimental and simulated data are compared for single-crystal silicon, showing the importance of accurate crystal orientation and many other corrections for obtaining good measurement statistics.

## 1. Introduction

Electron diffraction (ED) is currently enjoying increased attention and research activity due to its ability to work with crystallites that are far smaller than can be tackled by X-ray diffraction (XRD) (Gemmi *et al.*, 2019 ▸; Gruene *et al.*, 2018 ▸). Structural solution utilizing ED has dramatically increased since the turn of the century due to advances in computer control and detector development (Gemmi & Lanza, 2019*a* ▸) and the new methodologies that have been developed for structure solution are generally known by the term three-dimensional electron diffraction (3D-ED) (Gemmi *et al.*, 2019 ▸). Just as in XRD, these techniques measure the direction and integrated intensity of many Bragg-diffracted beams from a crystal, which are then processed to deduce a unit cell, given Miller indices *hkl* and observed intensities 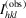 . These data are then used to produce a crystal model using structure solution methods. There are many differences between ED and XRD (Gemmi & Lanza, 2019*b* ▸), including very different wavelengths and damage mechanisms, but the principal one which affects diffracted intensities is the strength of the interaction, with electrons roughly 10 000 times more likely to be scattered than X-rays (Spence, 2017 ▸; Xu & Zou, 2019 ▸). It is this aspect which allows ED to outperform XRD at the nanoscale, but renders it unable to tackle macroscopic crystals. The methods are therefore complementary and together they make a powerful combination (Yun *et al.*, 2015 ▸).

Even at the nanoscale, multiple scattering is usual for electrons – it is essential to capture the interaction of a fast electron with even a single gold atom (Howie, 2014 ▸) – whereas single scattering usually dominates for XRD. Since structural refinement relies on minimizing the difference between 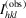 and calculated values 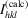 , it is therefore unsurprising that a fit to a single (kinematic) scattering model is poor for a method where multiple (dynamical) scattering dominates. Currently, some analyses of ED data for structural solution and refinement still use programs from the range of well established and relatively sophisticated XRD software, despite the vastly different scattering processes involved. As a result, structural solution statistics from 3D-ED often appear much worse than those of XRD, even though the structures obtained seem reliable. To develop the field of ED further, it is necessary to improve the quality of the fit by taking into account the differences between electron diffraction and other methods. As first demonstrated by Palatinus *et al.* (2015*a* ▸), and now firmly established by Klar *et al.* (2021 ▸), significant improvements can be obtained when dynamical electron scattering effects are considered.

The differences between electrons and X-rays when used for structure solution can be relatively subtle in data that only contain integrated intensities, where each reflection is measured over a time interval during which the diffracted beam passes completely through its Bragg condition. This type of data is found in continuous-rotation electron diffraction (cRED) experiments, where each data frame typically covers one or more degrees of crystal rotation. However, electron detector technology has seen a significant improvement in both quantum efficiency and speed in recent years (Faruqi & McMullan, 2018 ▸; Paterson *et al.*, 2020 ▸; Gruene & Mugnaioli, 2021 ▸), allowing ever greater amounts of data to be obtained. These fast pixelated detectors can provide data that have fine resolution both temporally and in scattering angle. cRED experiments may now have data frames covering a small fraction of a degree (Fröjdh *et al.*, 2020 ▸). The additional information in such fine-sliced data allows the detail of electron scattering processes to be observed more clearly and provides an opportunity to model them more comprehensively. In this work, we explore data taken with a crystal orientation resolution of ∼0.1°, in combination with Bloch-wave electron diffraction simulations. Our aim is to elucidate the most important experimental and modelling parameters that will be necessary in future electron diffraction methods.

The main reason for the continued adherence to a scattering model that is known to be inadequate for ED is the relative difficulty of calculation for dynamical scattering in comparison with the kinematic model. In both models, the starting point for calculation of the diffracted intensity for a reflection **g** = *hkl* is the structure factor *F*_*hkl*_, 

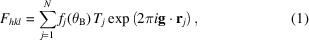

where *f*_*j*_(θ_B_) are the atomic scattering factors evaluated at the Bragg angle θ_B_, *T*_*j*_ are the thermal factors and **r**_*j*_ are the fractional atomic coordinates of the *j*th atom, the sum taken over all *N* atoms in the unit cell. In the kinematic model, it is commonly stated that the structure factor *F*_*hkl*_ is proportional to the amplitude of the diffracted beam, *i.e.* intensity is proportional to 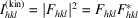 , where * indicates the complex conjugate. Using tabulated scattering factors equation (1) can be evaluated almost instantaneously on even the most basic computer. In comparison, modelling dynamical scattering for ED to obtain 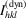 requires solving Schrödinger’s wave equation for an electron travelling through the crystal, usually done using either the Bloch-wave method or a multislice wave scattering/wave propagation calculation. The structure factor enters the Bloch-wave calculation as elements in the scattering matrix, which contains all excited **g** vectors and their differences, but is present only indirectly in a multislice calculation. These models, which require significant computing resources and may be cluster- or GPU-based, are widely used in more traditional ED work such as convergent-beam electron diffraction (CBED) (Tsuda & Tanaka, 1995 ▸; Spence, 1993 ▸; Zuo & Spence, 2013 ▸) and in the simulation of transmission electron microscopy (TEM) and scanning TEM (STEM) images.

Prior to the development of 3D-ED methods, an additional difficulty in ED resulted from the collection of static diffraction patterns, meaning that with parallel illumination almost all reflections were measured away from the Bragg condition. In this case, even minuscule changes in crystal orientation can produce large changes in diffracted intensity, and while dynamical modelling was possible (Jansen *et al.*, 1998 ▸) it was not straightforward. The introduction of beam precession (Vincent & Midgley, 1994 ▸) allowed the measurement of intensities integrated around a circular path in reciprocal space, which reduced the sensitivity to precise crystal orientation. However, dynamical modelling of intensities in individual ED patterns with beam precession then had to take account of the different integration path for each **g** vector (Sinkler & Marks, 2010 ▸; Dudka *et al.*, 2007 ▸). In order to solve a crystal structure in three dimensions, multiple precession ED measurements were obtained at different crystal orientations (Kolb *et al.*, 2007 ▸). This method has had great success but still requires corrections for integration of different **g** vectors (Palatinus *et al.*, 2015*b* ▸). In the current cRED method, integration is performed by rotation of the crystal in a similar manner to ‘X-ray rotation measurements’ (Arndt & Wonacott, 1977 ▸; Dauter, 1999 ▸). Extraction of integrated intensities in cRED data with, or without, beam precession is possible using the data reduction programs *Process Electron Tilt Series* or *PETS* (Palatinus, 2011 ▸; Palatinus *et al.*, 2015*a* ▸) and *PETS2* (Palatinus *et al.*, 2019 ▸), with structure solution and refinement with dynamical modelling using the programs *JANA* and *Dyngo* (Petříček *et al.*, 2014 ▸; Palatinus *et al.*, 2015*b* ▸, 2019 ▸; Klar *et al.*, 2021 ▸).

In any quantitative experiment, measurements and calculations must meet at some point and in XRD it is most convenient for that point to be the structure factor. The correspondence between model and experiment is usually captured using an *R* factor (see Section 3). Although it is often said that diffracted X-ray intensities from a crystal are proportional to |*F*_*hkl*_|^2^ [equation (1)], in reality things are not so simple and many other factors need to be accounted for, which depend on both the experiment being performed and the material. These factors are usually considered to be experimental and can be either measured or determined during refinement, but are separate from the crystal structure itself. As noted by Ladd & Palmer (1994 ▸), it can be helpful to list these corrections in the form of an equation, namely equation (2). This makes a distinction between raw experimental measurements of diffracted intensity 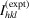 and the ‘observed’ intensities 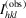 that are suitable for comparison with calculations 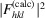 ,

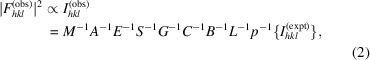

with corrections for polarization *p*, Lorentz factors *L*, background *B*, fluctuations in the incident beam intensity *C*, geometry *G*, scaling *S*, extinction *E*, absorption *A* and mosaicity *M*. Each correction in equation (2) will be different for each reflection *hkl* and should be considered a rather general operation when applied to a full diffraction data set. Furthermore if, like extinction in XRD, a correction is determined iteratively during refinement, it could be argued that it forms part of the scattering model and therefore should be applied to 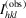 in equation (2) rather than 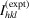 . For simplicity here, where we are concerned with the effect of these factors rather than their point of application during data processing, we consider any modification of data necessary to improve structure solution and refinement to be applied to the raw experimental data, resulting in a set of ‘observed’ intensities that depend only on the crystal structure and nothing else.

**Figure 1:**
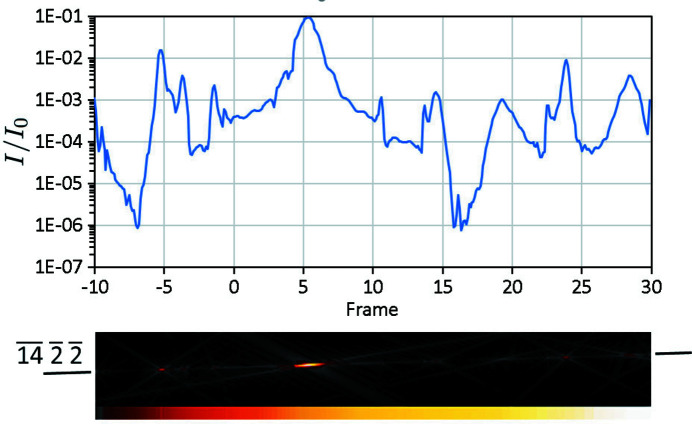
An illustration of the range of intensities that are obtained for the kinematically forbidden 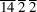 reflection in silicon with a thickness of 180 nm in a dynamical simulation. The image underneath shows the corresponding LACBED pattern (see section 3)

In XRD each reflection *hkl* has a single well defined integrated 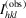 , so that if it is sampled multiple times, or there are symmetrically equivalent reflections, they can be merged into a single measurement with improved fidelity. However, in dynamical diffraction a reflection no longer has a single well defined intensity (Spence, 1993 ▸), as illustrated by Fig. 1 ▸. This shows a Bloch-wave simulation of a 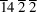 reflection from the silicon cRED data set (Section 3) which, although kinematically forbidden, has intensities up to 10% of the incident beam intensity where pathways for multiple allowed reflections exist. Although this is an extreme example, it is not uncommon for weak reflections to be affected in this way in ED. Thus an average intensity, taken either by merging multiple measurements, using symmetrically equivalent reflections or through the use of a precessed incident beam (Vincent & Midgley, 1994 ▸), will in general converge to some ill-defined value. Conversely, comparison of experimental data with a dynamical model in which each measurement is simulated individually gives a better match without any data merging (Palatinus *et al.*, 2015*b* ▸).

Before embarking on a structural refinement in which **r**_*j*_ and *T*_*j*_ in equation (1) are determined for each atom in the unit cell by minimizing the difference between observed and calculated intensities, the many corrections in equation (2) must be applied to optimize the experimental input 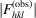 . At first sight, it might therefore appear that equation (1) deals with scattering theory while equation (2) deals only with experimental parameters, but this is not strictly correct – absorption and extinction, for example, are scattering effects that depend upon the sample. For dynamical electron diffraction, we must reconsider the validity of equation (2) to account for the differences in ED versus XRD experiments. For electrons, we can discard the polarization correction *p* since electron beams are unpolarized, but each of the other terms has an equivalent in ED. Thus, before presenting our results from a continuous rotation electron diffraction (cRED) measurement, we briefly discuss each in turn.

(i) *Lorentz corrections L*. Lorentz corrections ensure that a given reflection has the same integrated intensity in XRD irrespective of the way the crystal is rotated, *i.e.* they account for the different time spent in the vicinity of each Bragg condition during data collection. As purely geometric corrections, they apply equally to XRD and ED (Zhang *et al.*, 2010*b* ▸). If beam precession is employed in a set of ED patterns recorded from a static crystal, the circular path in reciprocal space taken by the direct beam must be taken into account (Gjønnes, 1997 ▸; Zhang *et al.*, 2010*a* ▸), but if data are collected with a continuously rotating crystal, beam precession adds no additional geometric correction factor to the integrated intensities (Palatinus *et al.*, 2019 ▸). In our cRED experiment, we measure the integrated intensity of each reflection individually from rocking curves in both experiment and simulation, and it is therefore possible to compare intensities without applying Lorentz corrections. However, to maintain equivalence with XRD refinement methods it is preferable to apply them, and we do so for both experiment and simulation.

(ii) *Geometry G*. Corrections for other geometric issues, such as variations in specimen height or rotation axis, are needed in XRD and also for ED (Palatinus *et al.*, 2019 ▸). These issues primarily affect a diffracted beam’s position, rather than the intensity that is our main interest here. It is of course important to ensure that the crystal of interest does not wander out of the electron beam as it is rotated (Cichocka *et al.*, 2018 ▸; Plana-Ruiz *et al.*, 2020 ▸). More importantly, since dynamical diffraction is exquisitely sensitive to geometry, the crystal orientation must be known to high precision. At crystal orientations where diffraction is strong, a good calculation of intensities in a cRED measurement using dynamical scattering requires an angular precision and accuracy better than 40 arcseconds (∼0.2 mrad). Previously, orientation refinement has been performed by optimizing the fit between experimental and calculated intensities, either integrated (Palatinus *et al.*, 2013 ▸) or pixel-wise for peaks detected on individual frames (Palatinus *et al.*, 2019 ▸). Here, we show that fine-sliced data allow a quicker and more straightforward orientation refinement to be performed, using the sequence of reflections as they appear during crystal rotation. The fine-sliced data approach also permits a measurement of the varying slew rate (Section 3). As a corollary of the requirement for high precision in crystal orientation, the angular range of the incident beam must also be taken into account; while parallel illumination is often assumed, in practice there is always some beam convergence or divergence that broadens the range of reciprocal space that is sampled.

(iii) *Background B*. In ED, corrections for background need to be considered from two main sources, (*a*) the detector and (*b*) non-Bragg scattered electrons (both elastic and inelastic). For (*a*), detector characteristics such as dark noise levels, quantum efficiency and linearity can be measured effectively; these corrections are necessary but straightforward. Unfortunately, (*b*) is rather more problematic. Amorphous material, *e.g.* the support film for the crystal, can create a nonlinear background (Tivol, 2010 ▸), but the sample itself also produces non-Bragg thermal diffuse scattering (TDS, caused by displacement of atoms from their mean positions by thermal vibrations) and inelastic scattering. Importantly, these electrons can be diffracted again by the crystal, producing a background that is highly structured, with Kikuchi lines and dynamical scattering effects (Eggeman & Midgley, 2012 ▸). Calculating this background is not a trivial exercise and generally requires a model of thermal vibrations (ideally, complete knowledge of the phonon spectrum) (Muller *et al.*, 2001 ▸; Kolb *et al.*, 2012 ▸) and a full quantum-mechanical description of inelastic processes (Forbes *et al.*, 2011 ▸), respectively. In a complete model of electron scattering these effects would be taken into account in the calculation of diffracted intensities to be compared against experiment. Currently, while some multislice simulation packages can do so (Allen *et al.*, 2015 ▸) none have yet been implemented for 3D-ED experiments. Here, we use a simple Bloch-wave model that neglects this ‘background’ intensity of diffuse scattering by the crystal.

(iv) *Absorption A*. This is another term which, strictly speaking, should be considered in scattering theory but in XRD its behaviour is simple enough for it to be corrected as an experimental variable. For the energies typical of ED (80–300 keV) and a thin specimen suitable for structure solution, true absorption of the electron beam does not happen to any appreciable extent. However, the attenuation of a Bragg reflection, as electrons are scattered into the diffuse background by TDS or inelastic interactions, has a very similar effect. In ED, TDS is enhanced significantly when the electrons are channelled along atom columns, particularly those with high atomic number (Hall & Hirsch, 1965 ▸). Thus, dark bands can be seen between low-index Bragg conditions in bright-field large-angle convergent-beam electron diffraction (LACBED) patterns [‘anomalous absorption’ (Hirsch *et al.*, 1965 ▸; Jordan *et al.*, 1991 ▸); see examples below]. This complicated behaviour means that in ED it is best dealt with in scattering theory, and should no longer be regarded as an experimental parameter. Both Bloch-wave and multislice models can account for this effect in ED.

(v) *Extinction E*. This is simply the X-ray term for dynamical diffraction effects. The underlying theory is very similar; the two-beam analysis by Darwin (1914*a* ▸,*b* ▸, 1922 ▸) for X-rays has many resemblances to the analysis of Howie and Whelan for electrons (Howie & Whelan, 1961 ▸; Hirsch *et al.*, 1965 ▸), so much so that they are sometimes referred to as the Darwin–Howie–Whelan model (James, 1990 ▸). The term ‘extinction’ refers to the transfer of intensity from the direct beam to a diffracted beam **g** and back again, as a function of crystal thickness; the distance over which this occurs is known as the extinction distance ξ_*g*_. In XRD this is usually dealt with during refinement and can be considered as a multiplicative correction factor that depends only upon the magnitude of *F*_*hkl*_ and the **g** vector (Becker & Coppens, 1974 ▸; Petříček *et al.*, 2014 ▸; Bourhis *et al.*, 2015 ▸). This simple approach can be used because X-ray extinction distances are usually much larger than the size of a crystallite. In ED, where extinction distances can be tens of nanometres and multiple beams are almost always excited, this is not the case. [In XRD, there is also ‘secondary’ extinction, which refers to the enhanced absorption of strong diffracted beams in large crystals (Ladd & Palmer, 1994 ▸), whose counterpart in ED is anomalous absorption, mentioned above]. Again, this correction should not be applied to ED data, but taken into account in the calculation of diffracted intensities.

(vi) *Scaling S*. This correction takes account of the varying proportion of the incident beam occupied by a crystal of irregular shape as it is rotated. This is certainly a correction that should be applied in principle in ED, although in practice extreme care must be taken not to confound it with simple loss of intensity in the direct beam due to a very strong diffracted beam, or the effect of ‘absorption’ due to TDS. ED has a potential advantage over XRD here, in that a crystal can be imaged directly, allowing scaling to be calculated from a series of images taken after a diffraction measurement (Plana-Ruiz *et al.*, 2020 ▸). Without these images, frame-by-frame scaling of intensities is difficult to calculate [*e.g.* the current implementation of dynamical refinement refines the scale as part of the refinement process rather than the data reduction process (Palatinus *et al.*, 2019 ▸)]. Here we propose a method using the direct beam intensity.

(vii) *Fluctuations in incident electron beam intensity C*. cRED measurements are in general very rapid; diffracted electron beam intensities are sufficiently high that they can be sampled to good precision even in a small fraction of a second, and the total time for data collection is often less than a minute. Variations in incident beam intensity on this timescale are negligible. Despite this, it is common to see significant changes in the direct 000 beam intensity in a cRED data set (see *e.g.* the video in the supporting information). This happens because electron diffraction is strong and the crystal occupies much, or all, of the incident electron beam. In ED, it is quite possible for a diffracted beam with a large structure factor to have a higher intensity than the direct beam. Nevertheless, this effect is not due to a change in incident beam intensity and therefore this correction is not appropriate for a cRED measurement.

(vii) *Mosaicity M*. Crystal imperfections, in the form of dislocations, low-angle grain boundaries and cracks, allow the Bragg condition to be satisfied over a wider range of angles than would be the case for a perfect crystal without strain. In XRD, the presence of these defects can be helpful in that they effectively break the crystal into a mosaic of small crystal blocks, reducing extinction effects significantly (Ladd & Palmer, 1994 ▸), although they can also produce broadening of diffracted beams. For high-energy electrons, with much smaller extinction distances, defects alter the diffracted intensity very strongly, allowing them to be visualized directly in diffraction-contrast TEM (Hirsch *et al.*, 1965 ▸; Williams & Carter, 2009 ▸). In ED they may have a significant effect on measured intensities that is too complicated to account for in any simple model and which will vary from one crystal to another in an unknown way. Currently, their effect is neglected completely and this is probably the best approach until the more tractable effects of dynamical diffraction are fully accounted for.

To summarize, in reconsidering equation (2) for dynamical ED structure solution or refinement, we find that some factors that can be separated from the scattering model and considered ‘experimental’ variables for XRD – *i.e.* absorption *A*, geometry *G* and extinction *E* – must instead be considered explicitly in the scattering model for ED. Other experimental factors – polarization *p*, incident beam intensity variations *C* and the complicated effects of mosaicity *M* – can probably be neglected at the current level of simulation fidelity, being either relatively unimportant or too difficult to tackle with current methods. Finally, the truly experimental corrections *L*, *B* and *S* that both XRD and ED hold in common must be taken into account, but the differences in Bragg angle and hardware mean that they are rather different for ED. One of the biggest changes in emphasis is that the point of contact between experimental measurements and modelled intensities in a dynamical refinement no longer has a simple and elegant interpretation relating to the structure factor.

In what follows, we use the reliability factor *R*_1_ (see Section S1.3 in the supporting information) as a metric to compare model and experiment, using the square root of the observed (corrected) intensity 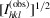 . Our discussion above justifies only corrections to the raw data for Lorentz factors *L*, background *B* and scaling *S*, 

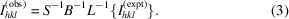




We compare 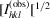 with the square root of the calculated ED integrated intensities 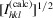 using a dynamical diffraction model in which extinction *X* and absorption *A* are implicit, resulting from the crystal itself. Dynamical integrated intensities 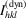 are a nonlinear function of crystal thickness *t*, geometry *G* and beam profile *P*. It should be borne in mind that the relationship between these values and the structure factor *F*_*hkl*_ of equation (1) is no longer straightforward. Since we apply Lorentz correction factors *L* to the simulation we distinguish between the raw simulation 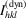 and corrected intensities 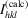 ,

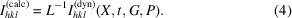




We now explore this approach using Bloch-wave simulations to compare kinematic and dynamical model fits to experimental data for a simple well known material. We examine data from a single crystal of silicon, ion milled to a thin foil. This almost perfect crystal shows strong dynamical scattering and is used to evaluate the improvement in fit using dynamical modelling, as well as the importance of correction factors applied to the raw data.

## 2. Experiment

### 2.1. Data collection

Experimental data were obtained using selected-area electron diffraction (SAED) with parallel beam illumination on a JEOL 2100 LaB_6_ transmission electron microscope operating at 200 kV. The sample was a defect-free single crystal milled using precision ion polishing (PIPS) Ar^+^ ions at 6 kV to electron transparency with surface damage minimized by final polishing at 0.5 kV, producing a (110) lamella with extensive thin parallel-sided regions. Diffraction patterns were produced using a strongly excited third condenser lens, giving close to parallel illumination and a selected-area aperture, and captured using a Gatan OneView camera recording continuously at 8× binning (4096 × 4096 → 512 × 512) at 75 frames per second. (Note that with 8× binning for data collection, the point spread function of the camera is negligible.) The crystal was much larger than the selected-area aperture and was rotated at maximum slew rate about the α-tilt axis over 143°, giving 1389 frames, each with a nominal crystal rotation of 0.1035° (*i.e.* all data collected in 18.5 s). The angular step per frame was determined by finding the lowest score parameter in *PETS* from the cylindrical projection plots.

### 2.2. Data reduction and dynamical simulations

We used *PETS* (Palatinus, 2011 ▸) to find the crystal orientation and unit cell, index reflections and give their rocking curves. The procedure followed sections 4.1–4.6 of Palatinus *et al.* (2019 ▸), which refers to the newer version *PETS2* (Palatinus *et al.*, 2019 ▸) but remains accurate for both versions. Bloch-wave simulations were performed using the code *Felix* (Beanland *et al.*, 2019 ▸) running on a high-performance computing cluster (typically 384 cores, completing a simulation in ∼30 s) using a Python script to extract data from the dyn.cif_pets file generated by *PETS* and write input files for *Felix*, *e.g.* setting the incident beam orientation with respect to the crystal. LACBED patterns of size 400 × 400 pixels were simulated over an angular range corresponding to 40 frames (4.14° or 72.26 mrad). The *x* axis of the image was taken to be along a direction perpendicular to the rotation axis. Successive simulations overlapped by ten frames in an attempt to ensure that each reflection was fully captured in a single simulation, an approach referred to as overlapping virtual frames (OVF) (Klar *et al.*, 2021 ▸). Seventy simulations were required in total to cover the full angular range of the cRED data.

## 3. Results

**Figure 2:**
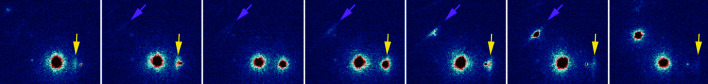
Seven consecutive frames from the Si cRED data set, showing Kikuchi lines as diffuse contrast passing through Bragg spots, marked by magenta and yellow arrows.

The Si cRED data contained 962 reflections up to 4 Å^−1^, with intensity over 6000 or 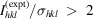 . Almost all reflections showed a single sharp peak as the crystal was rotated; only 27 had clear dynamical structure with multiple peaks in their rocking curves, obtained as an intensity given frame by frame as an output from *PETS*. Although these data were nominally background corrected [*B* ^−1^ in equation (3)], close inspection showed the tails of the rocking curves had a small non-zero average. An additional small background correction was therefore performed by subtracting a linear fit interpolated beneath the peak of each rocking curve (Fig. S1 in the supporting information). The origin of the small non-zero intensity when no peak was present is unclear, although we note that complex background from TDS and Kikuchi lines is readily apparent in the raw data, as shown in Fig. 2 ▸ (see also the animated data set in the supporting information).

The Lorentz correction *L* ^−1^ was then applied for each reflection **g** by taking the sum of counts in the background-subtracted rocking curve and multiplying by the change in deviation parameter per frame δ*s*_*g*_, 

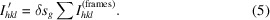




Since the silicon lamella was close to parallel-sided and much larger than the selected-area aperture, it filled the field of view completely throughout data collection and therefore no frame scaling correction *S* ^−1^ should be required. Nevertheless, it is useful to examine the total intensity falling on the detector and the intensity of the direct beam as the crystal is rotated, both shown in Fig. S2. The total intensity (blue line) shows a drop of approximately 10% when the crystal is perpendicular to the beam, close to zero α tilt. Since the silicon is not thick enough to absorb 200 kV electrons to any appreciable extent, and the short collection time precludes any significant change in incident beam intensity, the change in total intensity must be due to scattering of electrons to high angles outside the detector area. This observation remains unexplained. For the direct beam, each frame *z* was cropped to an *x* = 17 × *y* = 17 pixel image *I*(*x*, *y*, *z*) containing just the direct beam. An average beam profile was obtained by summing all frames and dividing by *n* = 1387, *i.e.*


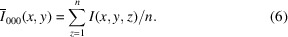

A normalized direct beam intensity 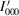 was then produced by dividing each cropped frame by 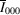 ,

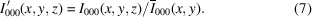

**Figure 3:**
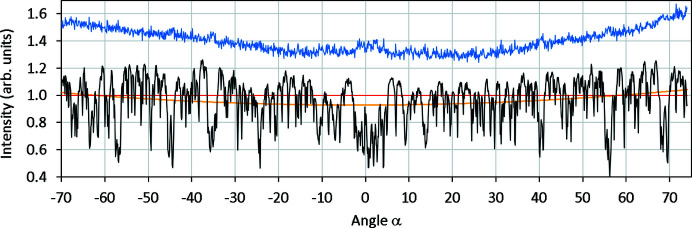
The blue line shows the total intensity in the Si cRED ED pattern as a function of goniometer angle α, normalized to an average of unity and offset by 0.4. The black line shows the relative direct beam intensity for the data, normalized to an average of unity (red line). See also Fig. S2.

Re-slicing this 17 × 17 × 1387 *xyz* data volume to 1387 × 17 × 17 *zyx*, and taking the average along *x*, gives a 1387 × 17 *yz* image that shows relative direct beam intensity as the crystal is rotated (Fig. S2, black line in Fig. 3 ▸). The trend in total intensity is also visible here (orange line). There is a range of approximately 10° at α = 0° where the relative intensity is depressed by ∼20%, but this is primarily due to channelling (see below); since this is calculated as part of the dynamical simulation it should not be compensated for by scaling. Most strikingly, there are many sharp and significant minima (up to 50% of the relative intensity). Closer examination shows each of these dark lines to occur when a diffraction condition is satisfied; they simply indicate a transfer of intensity from the direct beam to a diffracted beam. As seen in more detail in Fig. S2, Bragg conditions that are satisfied in only a few frames are visible as dark vertical bands, while Bragg conditions that pass through the direct beam slowly are visible as inclined dark lines. In the absence of any explanation of the broad variations in intensity and knowing that the crystal completely filled the selected-area aperture, no intensity scaling was applied.

**Figure 4:**
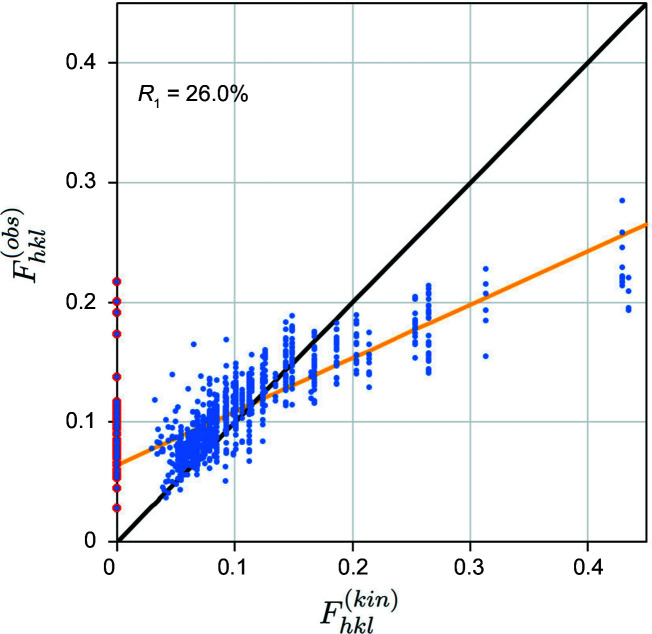
*R*_1_ calculation for cRED silicon data using the kinematic model [equation (1)] with *B* = 0.2. Kinematically forbidden reflections are highlighted in red. *R*_1_ = 26.0%

After applying corrections *B* ^−1^ and *L* ^−1^ and while discounting *S*, we perform an overall scaling to compare simulated and experimental intensities directly. Intensities are calculated with an incident beam intensity of unity and we multiply the corrected integrated observed intensities by a factor *K*,

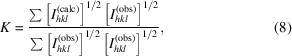

giving a set of integrated intensities 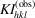 that can be compared with the simulation and which yield an *R* factor. We use the usual definitions, *e.g.* (Palatinus *et al.*, 2013 ▸)

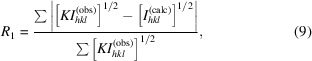




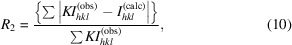




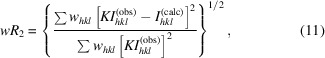

where the weights 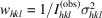 (Ladd & Palmer, 1994 ▸). We first consider kinematic intensities calculated using equation (1), taking the temperature factor to be *T*_*j*_ = exp[−*B*_*j*_sin^2^(θ)/λ^2^], where *B*_*j*_ is the Debye–Waller factor. We find a minimum *R*_1_ = 26.4% (*R*_2_ = 59.4%, *wR*_2_ = 56.2%) at *B* = 0, shown in Fig. 4 ▸ (X-ray refinements give *B* = 0.54 Å^2^; Többens *et al.*, 2001 ▸). In this plot the bold black line indicates *R*_1_ = 0 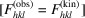 while the orange line is a least-squares linear fit to the data. It is quite clear that the fit of the kinematic model to the data is poor, not least because there are a number of kinematically forbidden reflections that have significant experimental intensities (highlighted in red), but also because many strong reflections ( 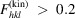 ) are in fact weaker than expected.

**Figure 5:**
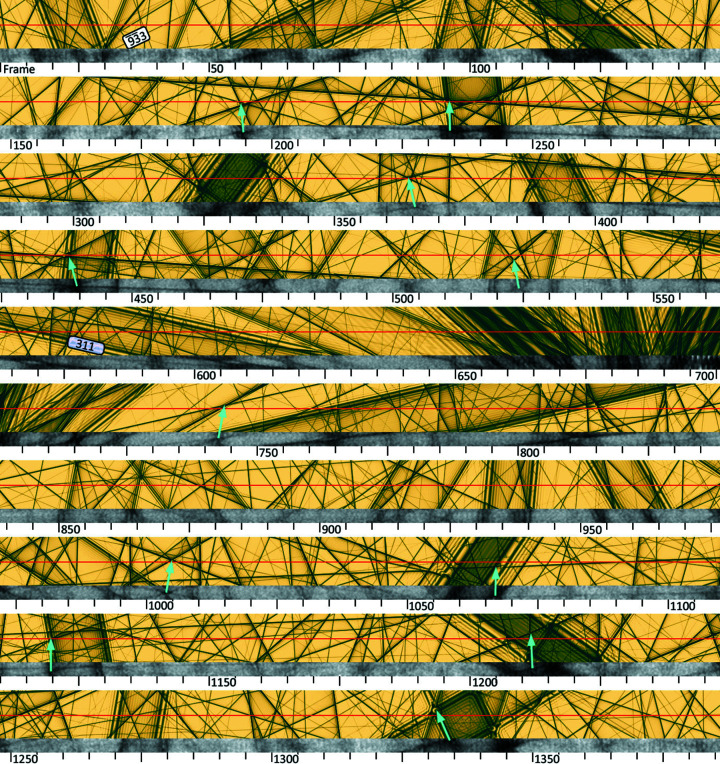
Dynamical simulation for the path of the 000 beam through reciprocal space (red line) in the silicon cRED data set, corresponding to 1387 frames (goniometer rotation of 143° about α). Frame numbers are indicated; note that one division = 5 frames = 0.5175° = 9 mrad. Each dark line corresponds to a Bragg condition for a diffracted beam, two of which ( 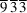 , frames 30–50, and 311, frames 545–590) are labelled. Below the simulation, the experimental intensity of the 000 beam is shown. Some features that are clearly present in both simulation and experiment are indicated by arrows. The [110] zone axis lies a few degrees below the beam path around frame 670.

The poor *R*_1_ seen in Fig. 4 ▸ is typical of many ED refinements using a kinematic model and we now turn to a dynamical one. We use an initial Debye–Waller factor *B* = 0.54 (Többens *et al.*, 2001 ▸) since the value obtained from the kinematic model is unphysical. A Bloch-wave simulation of seventy *Felix* 000 LACBED patterns, stitched together to make a continuous strip, is shown in Fig. 5 ▸. The frame numbers underneath correspond to experiment. In this image, a perfect plane-wave incident beam corresponds to a single point and the red line marks the nominal path traced by the direct beam through reciprocal space as the crystal is rotated. Each dark line in the simulation shows the location of a Bragg condition; when the direct beam lies on one of these lines a diffracted beam is produced, with an intensity that can be obtained from the corresponding point in the relevant dark-field LACBED pattern. The correspondence between this simulation and experiment can also be seen by converting the normalized experimental direct beam data volume to a 2D image, shown in greyscale below the simulation. Some features common to both experiment and simulation are marked by arrows.

**Figure 6:**
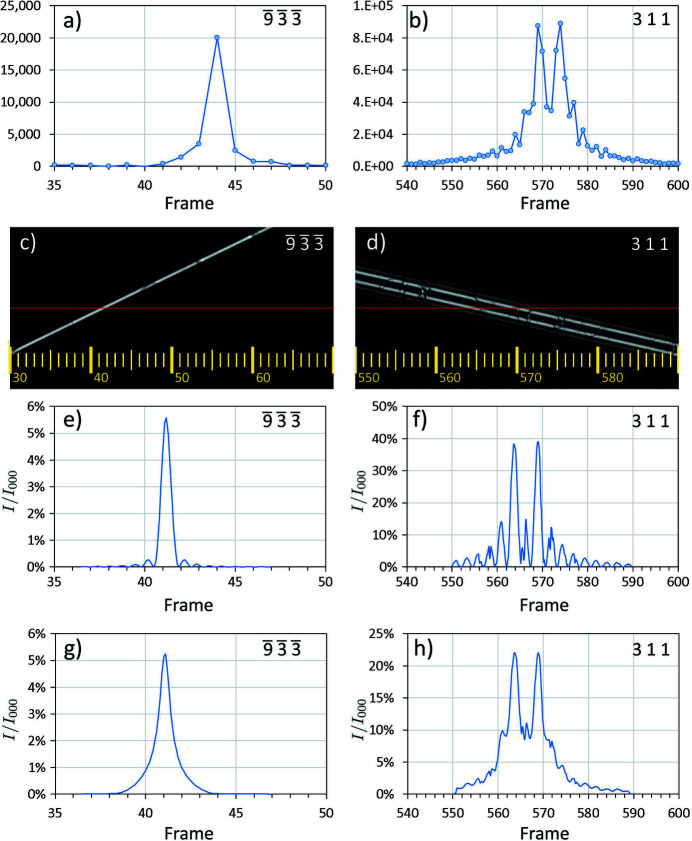
Two examples of silicon cRED rocking curves. (*a*) and (*b*) are experimental data. Most experimental rocking curves have a simple peak like (*a*) 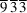 , while less than 3% show dynamical structure like (*b*) 311. (*c*) and (*d*) The corresponding dark-field LACBED simulations (specimen thickness 185 nm). The nominal beam path is a red line, with frame numbers in yellow. Intensity profiles along the red line give the rocking curves (*e*) and (*f*). The difference in frame numbers (*a*) to (*e*) and (*b*) to (*f*) is caused by a varying slew rate [Fig. 9(*b*)]. (*g*) and (*h*) Applying the angular spread of the incident beam (Fig. 10) as a convolution to the simulation gives simulated rocking curves that are a good match to experiment.

The simulated rocking curve for a reflection is given by the intensity along a line in its dark-field LACBED pattern, as shown in Fig. 6 ▸ for (*a*) a typical reflection with a single peak 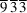 and (*b*) one with obvious dynamical structure 311. Integrated intensities 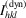 can be obtained from these simulated rocking curves in the same way as they are taken from experimental ones, although here there is no diffuse scattering and therefore no background correction. The incident beam intensity is fixed at unity so there is no scaling correction and only Lorentz corrections need to be applied.

**Figure 7:**
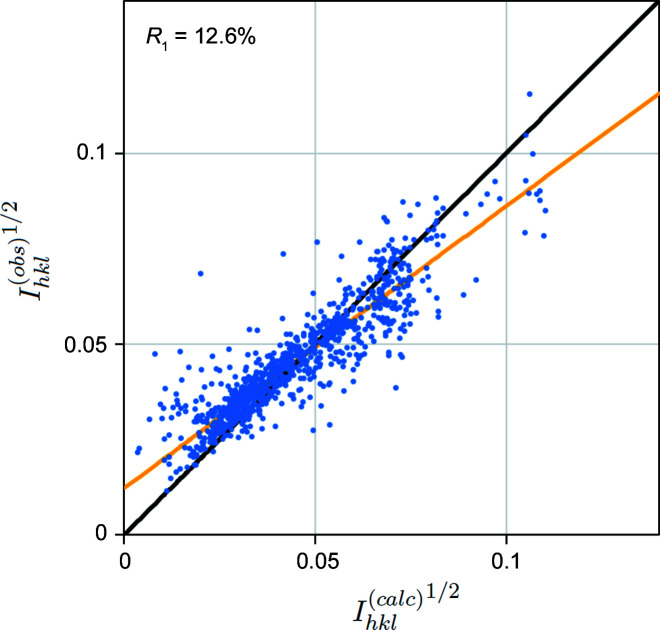
*R*_1_ calculation for cRED silicon data with a Bloch-wave model, using the nominal direct beam path (red line in Fig. 3 ▸) and a specimen thickness of 190 nm. The kinematically forbidden reflections identified in Fig. 4 ▸ now have non-zero values. The black line marks perfect correspondence (*R*_1_ = 0) and the orange line a least-squares linear fit. *R*_1_ = 12.6%

Kinematic intensities are independent of crystal thickness, but dynamic intensities can be very sensitive to it, particularly strong reflections with short extinction distances. It is therefore necessary to perform simulations for a range of thicknesses (see supplementary Sections S1.4 and 1.5). We may also expect the specimen thickness along the path of the electron beam to vary as the crystal is rotated, but this is ignored for the moment. The best *R*_1_ is obtained for a thickness of 190 nm, shown in Fig. 7 ▸. A very significant improvement over the kinematic model is apparent, with *R*_1_ = 12.6% (*R*_2_ = 23.8%, *wR*_2_ = 39.8%). The wide spread of intensities is no longer present, but there is still considerable scatter about the expected *R*_1_ = 0 line and the gradient of a linear fit is 0.74.

Dynamical simulations therefore clearly give a much better fit to experimental data, as may be expected. However, *R*_1_ is still relatively high in Fig. 7 ▸ and further improvements are possible by increasing the precision of the crystal orientation. The wide range of reciprocal space covered in the simulation allows the geometry to be optimized, as described below.

### 3.1. Orientation optimization *G*

For any given reflection in Fig. 5 ▸ its Bragg condition is satisfied, and a spot will appear in the SAED pattern, when the 000 beam sits on the corresponding dark line. The frame in which the maximum diffracted intensity appears is given by the crossing point of the Bragg condition and the red line. It is thus possible to obtain the sequence of reflections which appear in a cRED experiment, and the frame spacing between them, for any given direct beam path. Conversely, with knowledge of the frames in which diffracted maxima appear in an experiment we can find the corresponding path through reciprocal space.

**Figure 8:**
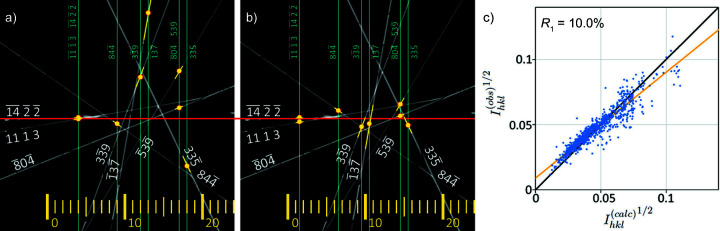
(*a*) The intersections with Bragg conditions for eight reflections in the first Si *Felix* simulation. Frame numbers are given at the bottom of the image and the frame in which each peak intensity is found is marked by a vertical blue line. The intersection of the blue line with its Bragg condition is marked by a yellow dot (yellow lines correspond to an error of ±0.5 frames). The horizontal red line marks the nominal beam path (output from *PETS*) and if the crystal orientation was correct the yellow dots would all lie on this line. (*b*) The best fit to a straight line is obtained by shifting the blue lines by −2.5 frames. (*c*) *R*_1_ calculation using an optimized beam path and a specimen thickness of 185 nm. *R*_1_ = 10.0%

**Figure 9:**
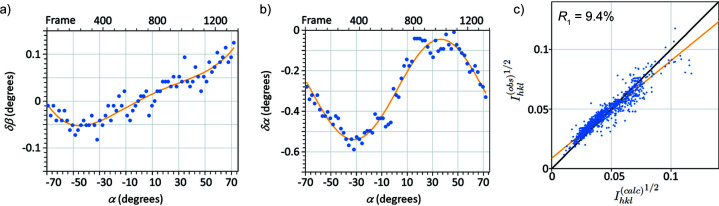
Corrections to the nominal direct beam path (red line in Fig. 5 ▸), (*a*) perpendicular to the line (β tilt) and (*b*) along the line (α tilt). The uncertainty in δα and δβ for each data point is ∼ ± 0.5 frames, *i.e.* 0.05°. (*c*) *R*_1_ calculation after correcting the integrated intensities for a varying slew rate gives *R*_1_ = 9.4% at a specimen thickness of 195 nm.

The effect of a slightly incorrect crystal orientation is shown in Fig. 8 ▸(*a*). In this simulated image each frame corresponds to a vertical stripe ten pixels wide and the nominal direct beam path runs horizontally through the centre, marked by a red line. Experimentally, the 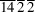 and 11 1 3 reflections were seen in frame 4, 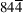 was seen in frame 9 *etc.*, as marked by blue vertical lines. The image is a superposition of eight dark-field LACBED patterns; each bright line corresponds to a diffracted beam (and to a dark line in the direct beam LACBED pattern, not shown). The intersection of each blue line and its corresponding diffraction condition is marked by a yellow dot and must lie on the beam path; yellow lines indicate an error of 0.5 frames. Clearly, these points do not correspond to the expected horizontal line through reciprocal space, indicating that the nominal crystal orientation is slightly in error. An optimized crystal orientation can be found by shifting the group of blue lines while maintaining their relative frame spacing (*e.g.* 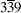 has an experimental peak intensity one frame before 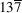 and three frames after 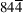 ). Shifting the set of blue lines by 2.5 frames to the left brings all points close to a horizontal line [Fig. 8 ▸(*b*)]. An optimized direct beam path was then calculated by fitting a smoothed curve to the best crystal orientation for each simulation (Fig. 9 ▸), for both changes about the rotation axis δα and perpendicular to it δβ, by least-squares fitting a horizontal line to the optimized set of intersection points. Rocking curves extracted from the Bloch-wave simulations using an optimized beam path give a significant improvement to *R*_1_ = 10.0% [*R*_2_ = 19.9%, *wR*_2_ = 30.2%, Fig. 8 ▸(*c*)], mainly by reducing the scatter in reflections with lower intensities. This can be understood by referring back to Fig. 1 ▸, which shows how large variations in intensity can be found in weak beams when they coincide with stronger beams. Optimization of the beam path is essential to capture these interactions correctly.

The resulting corrections are shown in Fig. 9 ▸. The actual path traced by the direct beam deviates vertically from the red line (*i.e.* about the β tilt axis) in Fig. 9 ▸ by a maximum of ten pixels (equivalent to one frame or 0.1°). There is a much larger correction needed along α, up to five frames [0.5°, Fig. 9 ▸(*b*)] and which varies through the data series, caused by a varying slew rate during rotation of the specimen. This varying slew rate is apparent in Fig. 5 ▸, where the features in the normalized experimental direct beam intensity are not found directly beneath their corresponding points in the simulation above. A changing slew rate also has an impact on integrated intensities, since the crystal is rotating more slowly or quickly through a diffraction condition than expected. Applying a slew rate correction to the simulated intensities gives a further improvement to *R*_1_ = 9.4% [*R*_2_ = 13.8%, *wR*_2_ = 28.7%, Fig.9 ▸(*c*)].

### 3.2. Correction for beam profile *P*

The sensitivity of dynamical electron diffraction to thickness is apparent in the *R*_1_ calculation for all integrated intensities (see Section S1.4). It is also very important for the fine structure of rocking curves of strongly dynamical interactions, which show fringes that change in size and number as a function of crystal thickness. It has long been known that crystal thickness can be measured to an accuracy of a nanometre or better using these features in CBED patterns (Kelly *et al.*, 1975 ▸; Allen, 1981 ▸). The fine structure of strongly dynamical rocking curves thus gives another way of measuring crystal thickness, which should match the minimum *R*_1_ for all reflections. These fringes can be seen clearly for the 311 reflection in Fig. 6 ▸. However, the features in Fig. 6 ▸(*f*) are noticeably sharper than the experimental rocking curve in Fig. 6 ▸(*b*) and this is due to the angular range of the incident electron beam. Additionally, in the optimized orientation of Fig. 8 ▸(*b*) the yellow dots do not lie precisely on a straight horizontal line. Both of these effects may be explained if the intersections are not points but have a finite size.

**Figure 10:**
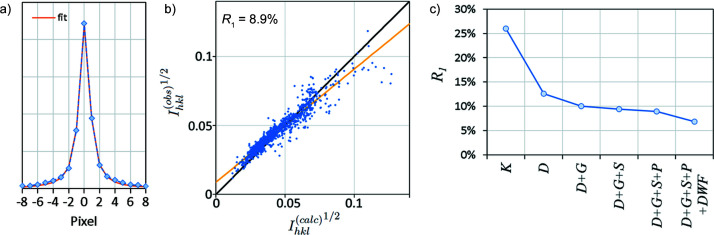
(*a*) The direct beam profile, obtained by averaging all frames in the Si cRED data and a Lorentzian fit. (*b*) *R*_1_ calculation after optimizing the geometry and correcting for the slew rate and beam profile (specimen thickness 185 nm). *R*_1_ = 8.9%. (*c*) *R*_1_ for the kinematic model *K* and initial dynamical model *D*, with geometry optimization *G*, corrections for slew rate *S* and beam profile *P*, and refinement of Debye–Waller factor *DWF*.

The incident electron beam is not a perfect plane wave because the crossover produced by the final condenser lens, which acts as an effective illumination source, is of finite size. We may approximate the angular profile of the incident beam by the intensity profile of the direct transmitted beam averaged through all frames, which is shown in Fig. 10 ▸(*a*) together with a fit to a Lorentzian profile. The fit is excellent and gives the FWHM of the direct beam as 0.037 Å^−1^ or 0.47 mrad (97 arcseconds). Thus, the integrated intensity is obtained from the simulated LACBED patterns not from a single row of pixels, but from multiple rows, each of which contributes in proportion with the beam profile of Fig. 10 ▸(*a*). This has the effect of changing the rocking curve profile, particularly when the line of the Bragg condition is more parallel to the beam path, and changing the integrated intensities because the intensity of a reflection varies along its Bragg condition. The most straightforward way of performing this correction is to apply the beam profile as a convolution to the simulations, which allows rocking curves to be extracted that are a good approximation to experiment, shown in Figs. 6 ▸(*g*) and 6 ▸(*h*). Integrated intensities obtained after applying this final correction give *R*_1_ = 8.9% [*R*_2_ = 18.1%, *wR*_2_ = 25.3%, Fig. 10 ▸(*b*)]. The largest scatter from the *R*_1_ = 0 line is now found in the highest intensity reflections.

**Figure 11:**
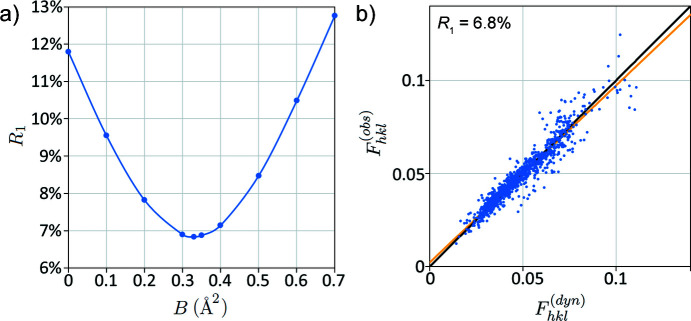
(*a*) *R*_1_ as a function of Debye–Waller factor *B*. (*b*) Optimized *R*_1_ calculation for *B* = 0.33, giving a best *R*_1_ = 6.8% at a specimen thickness of 185 nm.

Having applied all relevant corrections to the experimental data and optimized the simulation, we are finally in a position to perform a structural refinement. In silicon there is only one free parameter, the Debye–Waller factor *B*. Fig. 11 ▸ shows the variation in *R*_1_ with *B*, with a final *R*_1_ of 6.8% (*R*_2_ = 13.8%, *wR*_2_ = 23.6%) and a well defined best fit at *B* = 0.32Å^2^. This is rather lower than the X-ray value of *B* = 0.54Å^2^ (Többens *et al.*, 2001 ▸) and there is still noticeable scatter in the highest intensity measurements. These differences may be due to difficulties in background subtraction, which result from a limited number of intensity measurements in the *PETS* output (see Section S1.1).

In summary for these silicon cRED data, we have demonstrated that the poor *R*_1_ obtained using a kinematic diffraction model is not found when using a dynamical model with appropriate optimization and correction factors [Fig. 10 ▸(*c*)]. The final result (*R*_1_ < 7%) is still some way above those typical of X-ray diffraction (Ross *et al.*, 2014 ▸), indicating that there is still work to do in data processing or modelling for electron diffraction. The importance of careful correction is very clear from the observation that the improvement in *R*_1_ due to refinement of the temperature factor (Fig. 11 ▸) is of similar magnitude to improvements that result from optimization of the geometry [Fig. 10 ▸(*c*)]. Nevertheless, dynamical ED intensities are far more sensitive to structure (*i.e.* atomic coordinates) than temperature factors (Beanland *et al.*, 2021 ▸), which is reassuring for structure solution and consistent with the growing number of structures solved by ED. Further improvements may be possible with improved background subtraction, which may be incorrect for high-intensity reflections in this particular example due to a lack of data in the rocking curves.

## 4. Discussion and conclusions

In this work, we have established a protocol for dynamical modelling of fine-sliced cRED data, taking account of the corrections that should be applied in the case of electron diffraction, equivalent to those applied to X-ray data. These corrections rely on the ability to extract rocking curves from experimental data, *i.e.* having a large number of frames collected at small angular increments. We expect that this approach will become widespread as detector technology continues to improve. Our results show similar improvements to those seen by Palatinus *et al.* (2013 ▸, 2015*b* ▸) and Klar *et al.* (2021 ▸), *i.e.* dynamical modelling of cRED data has a very significant impact on the quality of fit, reducing *R*_1_ by almost 20% in the silicon example chosen here. Nevertheless, this is a particularly simple material with very high perfection and many systematic absences. Equivalent improvements may not be found for more interesting (complex) materials, particularly if they have poorer crystallinity, exhibit strong inelastic scattering as seen in organic materials (Latychevskaia & Abrahams, 2019 ▸) and are not parallel-sided lamellae.

Several improvements can be made from this attempt at dynamical modelling, not least the need for significant computing resources. Each set of 70 simulations calculated 5.6 × 10^6^ incident beam orientations, producing a 400 × 400 pixel LACBED pattern for each of the 962 diffracted beams. The large range of reciprocal space covered in each LACBED pattern, together with knowledge of which frame each reflection was seen in experimentally, allowed precise correction of the crystal orientation, but the area of reciprocal space covered could be reduced by a factor of >40 if this optimization were first performed by direct calculation of the positions of the different Bragg conditions. To allow rocking curves to be captured fully, each simulation overlapped with the next, meaning that every incident beam orientation was simulated twice, something easily avoided if a single simulation (*e.g.* 20 × 14 000 pixels) is calculated instead. Rocking curves and integrated intensities could be output directly, rather than extracted from these simulated data using Python scripts. In *Felix*, the Bloch-wave calculation is optimized by careful choice of the diffracted beams included (Zuo & Weickenmeier, 1995 ▸; Chuvilin & Kaiser, 2005 ▸), but the time required remains ∝ *N* ^3^, where *N* is the number of beams (Yang *et al.*, 2017 ▸). No attempt was made to optimize this parameter and all simulations were run with *N* = 200 from a beam pool of ∼800 in each simulation. If all such improvements were to be implemented it seems reasonable to expect a full set of 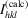 to be obtained in seconds. This would then allow dynamical refinement of crystal structure in reasonable times.

Improvements in simulation fidelity are also possible. The simple Bloch-wave calculation used here assumes that the surface normal is parallel to the incident beam direction, *i.e.* it does not properly account for continuity of the electron wavefunction at the entrance and exit surfaces of a tilted crystal. This is obviously not correct for a specimen tilted by up to 70°. Furthermore, as the crystal rotates, the thickness of material transited by the electron beam changes. Interestingly, we found that simulated rocking curves for a single thickness gave a good match to experiment across the full data set (Section S1.5), and this thickness agreed with the minimum *R*_1_ obtained from the integrated intensities. Incorporating a change in crystal thickness *t* corresponding to that expected for a parallel-sided slab gave no improvement in comparison with a single thickness for the complete data set, although large corrections should only occur at very high tilts ( 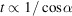 ). We expect a more correct model would yield further improvements in *R*_1_. Finally, some experimental rocking curves suffered from poor background correction (Section S1.1). While improved background subtraction may be possible in *PETS2*, the presence of diffuse scattering, modulated by strong Kikuchi lines, may still be a significant factor that prevents a good measurement of the intensity.

In conclusion, dynamical modelling of cRED data has a significant impact on the quality of the fit. Improvements are still required before the fit metrics for electron diffraction equal those of X-ray diffraction, but there seems to be no fundamental reason why they cannot be achieved. This is encouraging for the future development and application of 3D-ED techniques to structural solution in a wide range of applications.

## Supplementary Material

Additional figures. DOI: 10.1107/S2052252522011290/vq5003sup1.pdf

Experimental video of Si. DOI: 10.1107/S2052252522011290/vq5003sup2.avi

## References

[bb2] Allen, L., D’Alfonso, A. J. & Findlay, S. (2015). *Ultramicroscopy*, **151**, 11–22. 10.1016/j.ultramic.2014.10.011 25467859

[bb3] Allen, S. M. (1981). *Philos. Mag. A*, **43**, 325–335.

[bb1] Arndt, U. W. & Wonacott, A. J. (1977). Editors. *The Rotation Method in Crystallography: Data Collection from Macromolecular Crystals.* Amsterdam: North-Holland.

[bb4] Beanland, R., Evans, K., Römer, R. A. & Hubert, A. J. M. (2019). *Felix: Bloch-wave Method Diffraction Pattern Simulation Software.* https://github.com/WarwickMicroscopy/Felix.

[bb5] Beanland, R., Smith, K., Vaněk, P., Zhang, H., Hubert, A., Evans, K., Römer, R. A. & Kamba, S. (2021). *Acta Cryst.* A**77**, 196–207. 10.1107/S2053273321001546 PMC8127389 33944798

[bb6] Becker, P. J. & Coppens, P. (1974). *Acta Cryst.* A**30**, 129–147.

[bb7] Bourhis, L. J., Dolomanov, O. V., Gildea, R. J., Howard, J. A. K. & Puschmann, H. (2015). *Acta Cryst.* A**71**, 59–75. 10.1107/S2053273314022207 PMC4283469 25537389

[bb8] Chuvilin, A. & Kaiser, U. (2005). *Ultramicroscopy*, **104**, 73–82. 10.1016/j.ultramic.2005.03.003 15935917

[bb9] Cichocka, M. O., Ångström, J., Wang, B., Zou, X. & Smeets, S. (2018). *J. Appl. Cryst.* **51**, 1652–1661. 10.1107/S1600576718015145 PMC6276279 30546290

[bb10] Darwin, C. G. (1914*a*). *London, Edinb. Dubl. Philos. Mag. J. Sci.* **27**, 315–333.

[bb11] Darwin, C. G. (1914*b*). *London, Edinb. Dubl. Philos. Mag. J. Sci.* **27**, 675–690.

[bb12] Darwin, C. G. (1922). *London, Edinb. Dubl. Philos. Mag. J. Sci.* **43**, 800–829.

[bb13] Dauter, Z. (1999). *Acta Cryst.* D**55**, 1703–1717. 10.1107/s0907444999008367 10531520

[bb14] Dudka, A., Avilov, A. & Nicolopoulos, S. (2007). *Ultramicroscopy*, **107**, 474–482. 10.1016/j.ultramic.2006.03.009 17222976

[bb15] Eggeman, A. S. & Midgley, P. A. (2012). *Advances in Imaging and Electron Physics*, edited by P. W. Hawkes, pp. 1–63. Amsterdam: Elsevier.

[bb16] Faruqi, A. & McMullan, G. (2018). *Nucl. Instrum. Methods Phys. Res. A*, **878**, 180–190.

[bb17] Forbes, B. D., D’Alfonso, A. J., Findlay, S. D., Van Dyck, D., LeBeau, J. M., Stemmer, S. & Allen, L. J. (2011). *Ultramicroscopy*, **111**, 1670–1680. 10.1016/j.ultramic.2011.09.017 22088442

[bb18] Fröjdh, E., Wennmacher, J. T. C., Rzepka, P., Mozzanica, A., Redford, S., Schmitt, B., van Bokhoven, J. A. & Gruene, T. (2020). *Crystals*, **10**, 1148.

[bb19] Gemmi, M. & Lanza, A. E. (2019*a*). *Acta Cryst.* B**75**, 495–504. 10.1107/S2052520619007510 32830707

[bb20] Gemmi, M. & Lanza, A. E. (2019*b*). *Acta Cryst.* B**75**, 495–504. 10.1107/S2052520619007510 32830707

[bb21] Gemmi, M., Mugnaioli, E., Gorelik, T. E., Kolb, U., Palatinus, L., Boullay, P., Hovmöller, S. & Abrahams, J. P. (2019). *ACS Central Sci.*, **5**, 1315–1329. 10.1021/acscentsci.9b00394 PMC6716134 31482114

[bb22] Gjønnes, K. (1997). *Ultramicroscopy*, **69**, 1–11.

[bb23] Gruene, T. & Mugnaioli, E. (2021). *Chem. Rev.* **121**, 11823–11834. 10.1021/acs.chemrev.1c00207 PMC8517952 34533919

[bb24] Gruene, T., Wennmacher, J. T. C., Zaubitzer, C., Holstein, J. J., Heidler, J., Fecteau-Lefebvre, A., De Carlo, S., Müller, E., Goldie, K. N., Regeni, I., Li, T., Santiso-Quinones, G., Steinfeld, G., Handschin, S., van Genderen, E., van Bokhoven, J. A., Clever, G. H. & Pantelic, R. (2018). *Angew. Chem. Int. Ed.* **57**, 16313–16317. 10.1002/anie.201811318 PMC6468266 30325568

[bb25] Hall, C. R. & Hirsch, P. B. (1965). *Proc. R. Soc. London Ser. A*, **286**, 158–177.

[bb26] Hirsch, P. B., Howie, A., Nicholson, R. B., Pashley, D. W. & Whelan, M. J. (1965). *Electron Microscopy of Thin Crystals.* London: Butterworth.

[bb27] Howie, A. (2014). *J. Phys. Conf. Ser.* **522**, 012001.

[bb28] Howie, A. & Whelan, M. J. (1961). *Proc. R. Soc. London Ser. A*, **263**, 217–237.

[bb29] James, R. (1990). PhD thesis, University of Bath, UK.

[bb30] Jansen, J., Tang, D., Zandbergen, H. W. & Schenk, H. (1998). *Acta Cryst.* A**54**, 91–101.

[bb31] Jordan, I. K., Rossouw, C. J. & Vincent, R. (1991). *Ultramicroscopy*, **35**, 237–243.

[bb32] Kelly, P., Jostsons, A., Blake, R. & Napier, J. (1975). *Phys. Status Solidi A*, **31**, 771–780.

[bb33] Klar, P., Krysiak, Y., Xu, H., Steciuk, G., Cho, J., Zou, X. & Palatinus, L. (2021). ChemRxiv: https://doi.org/10.26434/chemrxiv-2021-4jh14.

[bb34] Kolb, U., Gorelik, T., Kübel, C., Otten, M. & Hubert, D. (2007). *Ultramicroscopy*, **107**, 507–513. 10.1016/j.ultramic.2006.10.007 17234347

[bb35] Kolb, U., Shankland, K., Meshi, L., Avilov, A. S. & David, W. I. F. (2012). Editors. *Uniting Electron Crystallography and Powder Diffraction.* Dordrecht: Springer.

[bb36] Ladd, M. F. C. & Palmer, R. A. (1994). *Structure Determination byX-ray Crystallography.* New York: Plenum Press.

[bb37] Latychevskaia, T. & Abrahams, J. P. (2019). *Acta Cryst.* B**75**, 523–531. 10.1107/S2052520619009661 PMC6690131 32830710

[bb38] A. Muller, D., Edwards, B., J. Kirkland, E. & Silcox, J. (2001). *Ultramicroscopy*, **86**, 371–380. 10.1016/s0304-3991(00)00128-5 11281157

[bb39] Palatinus, L. (2011). *PETS: Program for Analysis of Electron Diffraction Data.* Institute of Physics of the Czech Academy of Sciences, Prague, Czech Republic.

[bb40] Palatinus, L., Brázda, P., Jelínek, M., Hrdá, J., Steciuk, G. & Klementová, M. (2019). *Acta Cryst.* B**75**, 512–522. 10.1107/S2052520619007534 32830709

[bb41] Palatinus, L., Corrêa, C. A., Steciuk, G., Jacob, D., Roussel, P., Boullay, P., Klementová, M., Gemmi, M., Kopeček, J., Domeneghetti, M. C., Cámara, F. & Petříček, V. (2015*a*). *Acta Cryst.* B**71**, 740–751. 10.1107/S2052520615017023 26634732

[bb42] Palatinus, L., Jacob, D., Cuvillier, P., Klementová, M., Sinkler, W. & Marks, L. D. (2013). *Acta Cryst.* A**69**, 171–188. 10.1107/S010876731204946X 23403968

[bb43] Palatinus, L., Petříček, V. & Corrêa, C. A. (2015*b*). *Acta Cryst.* A**71**, 235–244. 10.1107/S2053273315001266 25727873

[bb44] Paterson, G. W., Lamb, R. J., Ballabriga, R., Maneuski, D., O’Shea, V. & McGrouther, D. (2020). *Ultramicroscopy*, **210**, 112917. 10.1016/j.ultramic.2019.112917 31841837

[bb45] Petříček, V., Dušek, M. & Palatinus, L. (2014). *Z. Kristallogr. Cryst. Mater.* **229**, 345–352.

[bb46] Plana-Ruiz, S., Krysiak, Y., Portillo, J., Alig, E., Estradé, S., Peiró, F. & Kolb, U. (2020). *Ultramicroscopy*, **211**, 112951. 10.1016/j.ultramic.2020.112951 32036199

[bb47] Ross, K. C., Petrus, J. A. & McDonald, A. M. (2014). *Powder Diffr.* **29**, 337–345.

[bb48] Sinkler, W. & Marks, L. D. (2010). *Z. Kristallogr.* **225**, 47–55.

[bb49] Spence, J. (2017). *Struct. Dyn.* **4**, 044027. 10.1063/1.4984606 PMC5453805 28653018

[bb50] Spence, J. C. H. (1993). *Acta Cryst.* A**49**, 231–260.

[bb51] Tivol, W. F. (2010). *Microscopy Today*, **18**(4), 22–28.

[bb52] Többens, D., Stüßer, N., Knorr, K., Mayer, H. & Lampert, G. (2001). *Mater. Sci. Forum*, **378–381**, 288–293.

[bb53] Tsuda, K. & Tanaka, M. (1995). *Acta Cryst.* A**51**, 7–19.

[bb54] Vincent, R. & Midgley, P. (1994). *Ultramicroscopy*, **53**, 271–282.

[bb55] Williams, D. B. & Carter, C. B. (2009). *Transmission Electron Microscopy: A Textbook for Materials Science.* New York: Springer.

[bb56] Xu, H. & Zou, X. (2019). *Science*, **364**, 632–633. 10.1126/science.aax5385 31097652

[bb57] Yang, Y., Yang, Q., Huang, J., Cai, C. & Lin, J. (2017). *Micron*, **100**, 73–78. 10.1016/j.micron.2017.04.007 28527398

[bb58] Yun, Y., Zou, X., Hovmöller, S. & Wan, W. (2015). *IUCrJ*, **2**, 267–282. 10.1107/S2052252514028188 PMC4392419 25866663

[bb59] Zhang, D., Grüner, D., Oleynikov, P., Wan, W., Hovmöller, S. & Zou, X. (2010*a*). *Ultramicroscopy*, **111**, 47–55. 10.1016/j.ultramic.2010.09.008 21051145

[bb60] Zhang, D., Oleynikov, P., Hovmöller, S. & Zou, X. (2010*b*). *Z. Kristallogr.* **225**, 94–102.

[bb61] Zuo, J. & Spence, J. (2013). *Electron Microdiffraction.* New York: Springer Science & Business Media.

[bb62] Zuo, J. & Weickenmeier, A. (1995). *Ultramicroscopy*, **57**, 375–383.

