# Supplementary material for: Modelling fine-sliced three dimensional electron diffraction data with dynamical Bloch-wave simulations
Source: IUCrJ. 2023 Jan 1;10(Pt 1):118–30. doi: 10.1107/S2052252522011290 (PMC9812222; doi:10.1107/S2052252522011290)
Supplement: Supplementary file 1 [file m-10-00118-sup1.pdf]

IUCrJ

Volume 10 (2023)

Supporting information for article:

Modelling fine-sliced three dimensional electron diffraction data with dynamical Bloch-wave simulations

Anton Cleverley and Richard Beanland

Supplemental Material for

Modelling fine-sliced three dimensional electron diffraction data with dynamical Bloch-wave simulations

S1.1. Experimental rocking curves and background subtraction

Four examples of rocking curves from the silicon cRED data produced by PETS, and subsequent background subtraction, are shown in Fig. S1. The red line is a linear least-squares fit to data points outside the peak. The raw integrated intensity is the sum of the green bars. For some strong reflections, such as the 111 peak, the data points in the rocking curve may not extend sufficiently to allow a good fit to the background, leading to a systematic underestimation of integrated intensity. Although not optimised in this data analysis, it is possible to increase the width of the rocking curve extracted by *PETS2* by modifying the mosaicity and rocking curve width parameters. It should thus be possible to extract the data in a way that the problem with insufficient`tails' of the rocking curves is avoided.

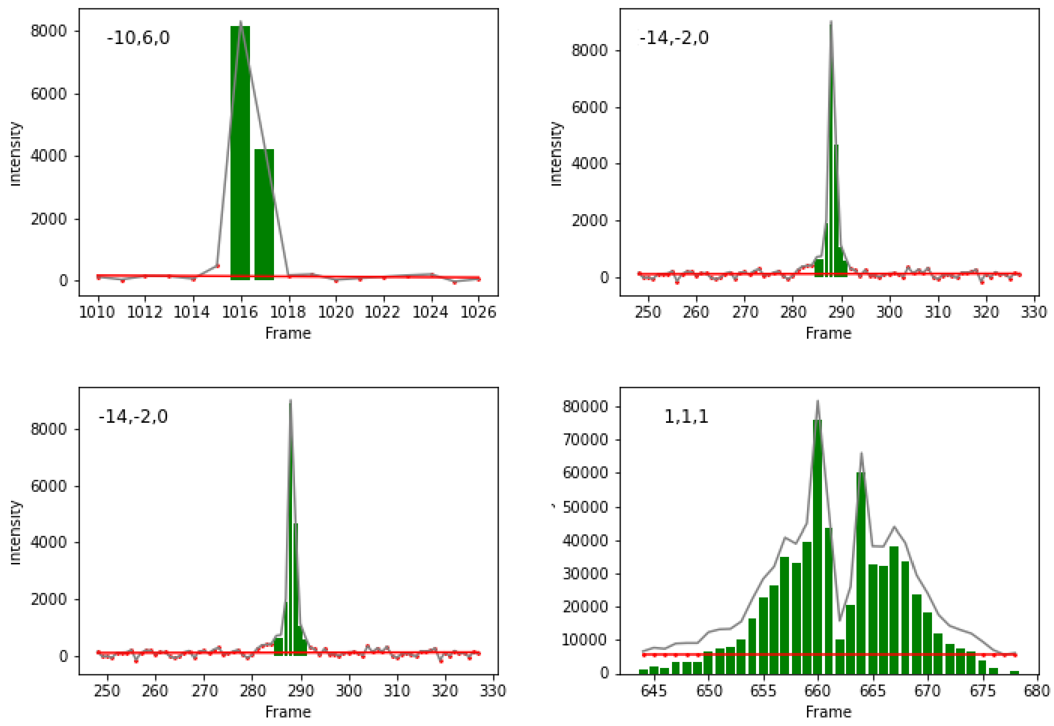

Fig. S1. Background subtraction for four rocking curves in the Si cRED data set. The line gives the frame by frame output intensity from PETS and the horizontal red line marks the measured background. Green bars show the intensity after background subtraction.

S1.2. Direct beam intensity

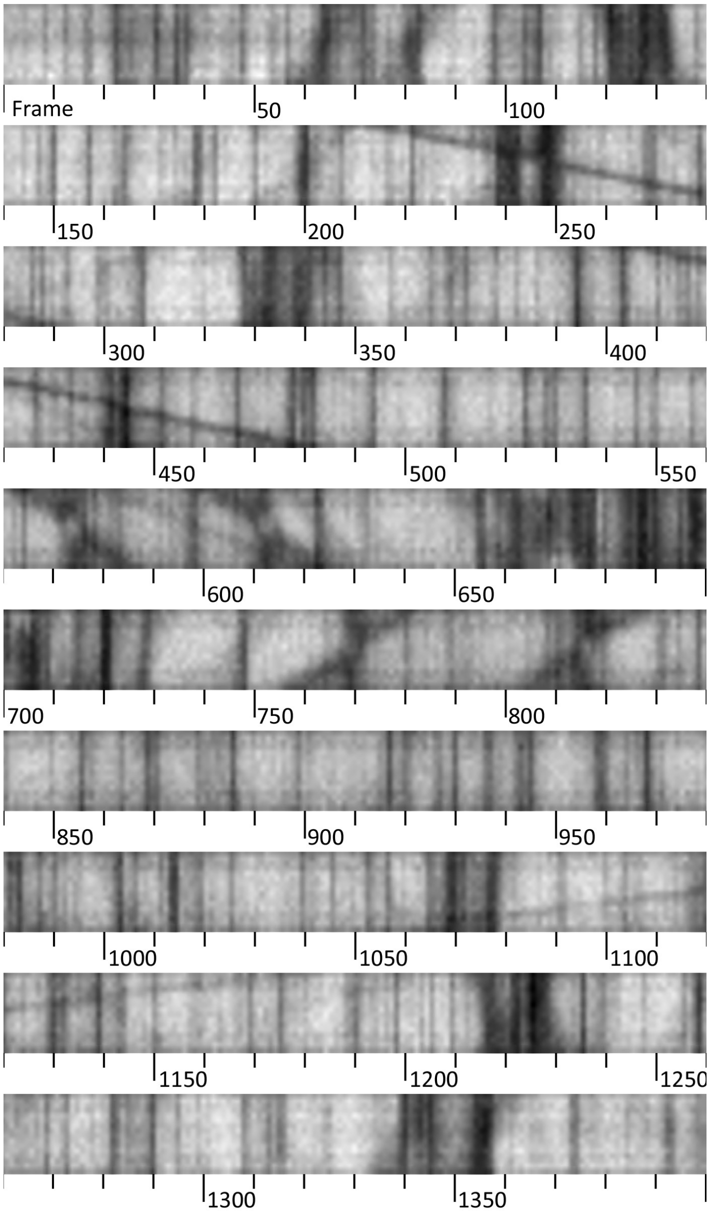

Fig. S2. The relative direct beam intensity, obtained by cropping the stack of $n = 1389$ frames to just the direct beam, producing an average of the beam profile by summing all frames and dividing by n, and then dividing each image in the stack by this average. The stack of frames is then re-sliced to view from the side. Deficits in the direct beam, caused by each Bragg condition that is passed through as the goniometer rotates, are visible as dark lines.

S1.3. Reliability factor R_1 calculation and sensitivity to B in the kinematic model

The usual equation for the reliability factor R_1 is

$$R_1 = \frac{\left(\sum ||F_{hkl}^{(obs)}| - |F_{hkl}^{(calc)}||\right)}{\sum |F_{hkl}^{(obs)}|}, \tag{S12}$$

where the sums are taken over all observed reflections. We use an equivalent version here for dynamical refinement, with $|F|$ replaced by $I^{1/2}$, i.e.

$$R_1 = \frac{\left(\sum |(I_{hkl}^{(obs)})^{1/2} - (I_{hkl}^{(calc)})^{1/2}|\right)}{\sum (I_{hkl}^{(obs)})^{1/2}}, \tag{S13}$$

The quality of fit can be seen by plotting $F_{hkl}^{(obs)}$ against $F_{hkl}^{(calc)}$ as shown in Fig. S3.

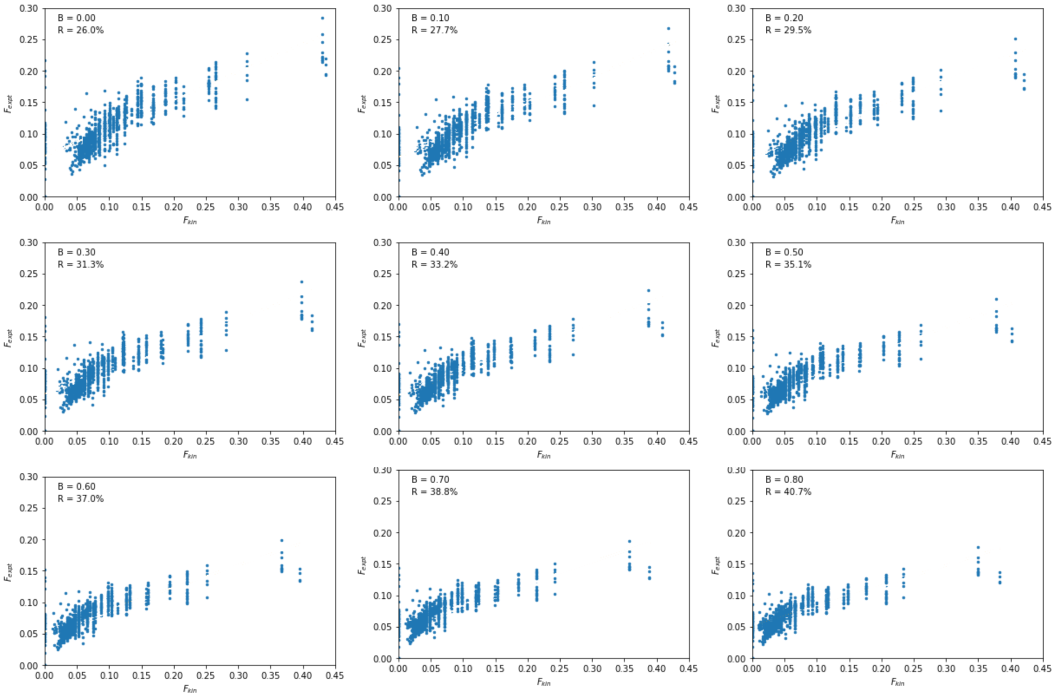

Fig. S3. R_1 calculation as a function of B in the kinematic model. The lowest R is found at $B = 0$.

S1.4. Dynamical R-factors as a function of thickness

Since diffracted intensities change significantly as a function of specimen thickness in a dynamical model, each specimen thickness has a different R_1. This is illustrated below in Figs. S4 and S5 for Bloch-wave simulations corresponding to the nominal beam path in Fig. 5.

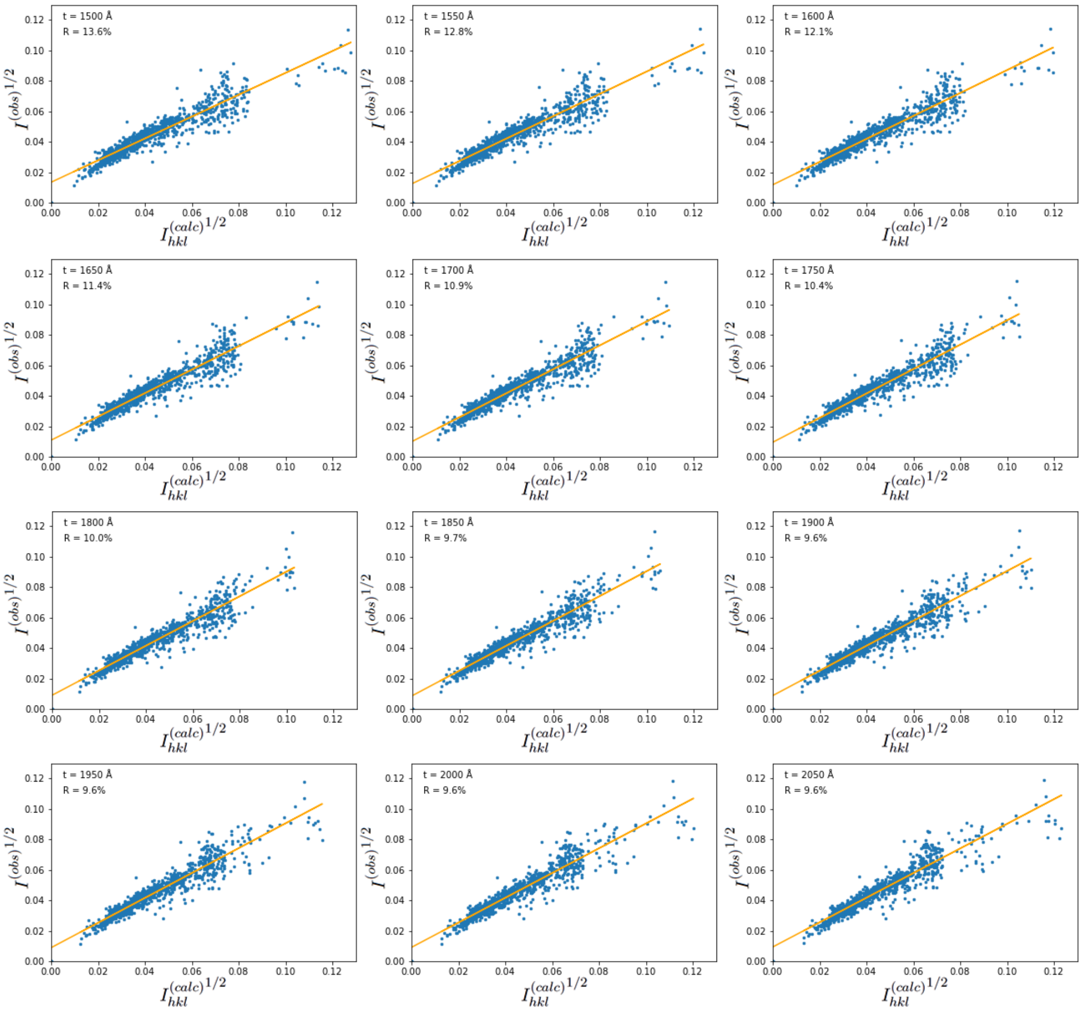

Fig. S4. R_1 calculation as a function of specimen thickness t in the dynamic model, with the nominal beam path given by $PETS$. Orange line is a least-squares linear fit.

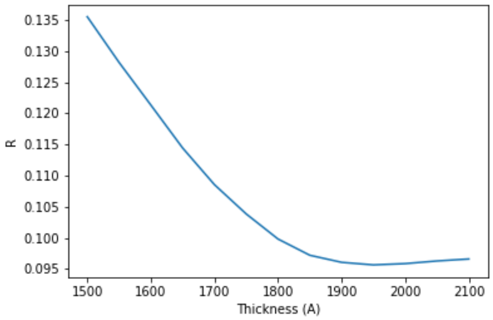

Fig. S5. R_1 as a function of specimen thickness t in the dynamic model, with an optimised beam path and beam profile convolution.

S1.5. Dynamical rocking curves as a function of thickness

The fine structure of rocking curves obtained from strongly dynamical reflections is very sensitive to specimen thickness, as exemplified here by Si 311. The simulated rocking curves have been convolved with the experimentally measured beam profile.

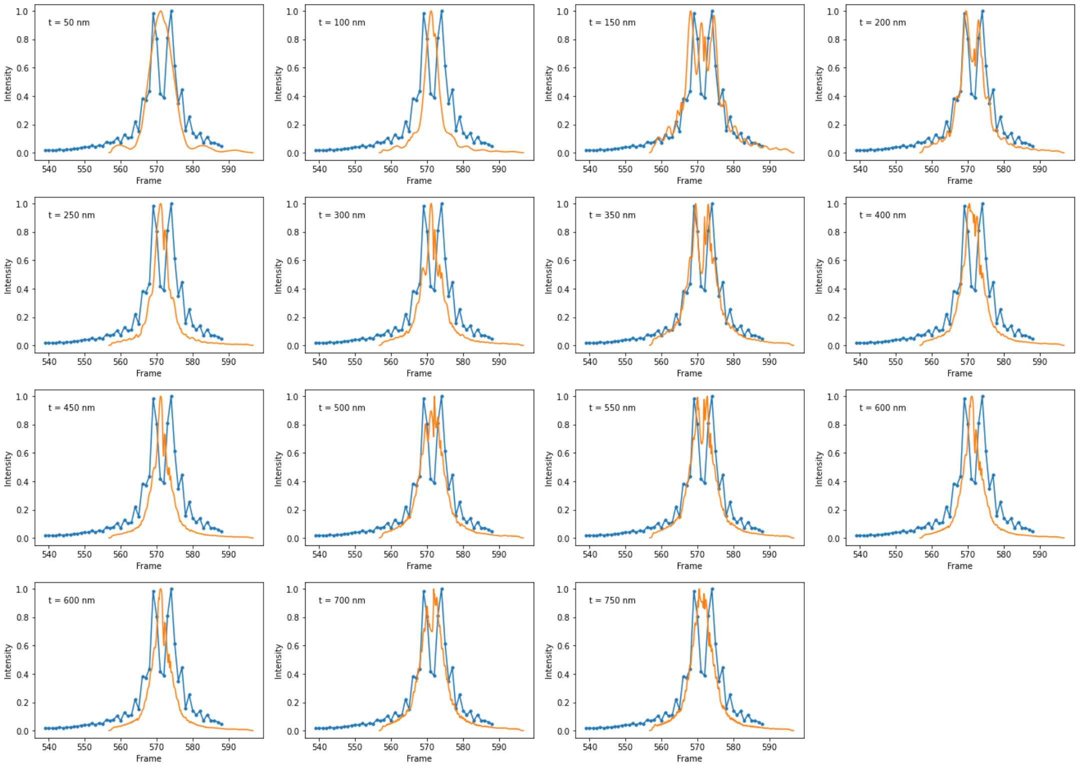

Fig. S6. Comparison of the experimental Si 311 rocking curve (blue) and simulations at a variety of thicknesses (orange).

S1.6. R factor as a function of B for optimised simulations

R_1 is sensitive to thermal vibrations of atoms in the dynamical model, as shown in the plots of ${I_{hkl}^{(obs)}}^{1/2}$ against ${I_{hkl}^{(calc)}}^{1/2}$ for different values of the Debye-Waller factor B. The systematic underestimation of strong reflections (S1.1) may lead to an underestimation of B.

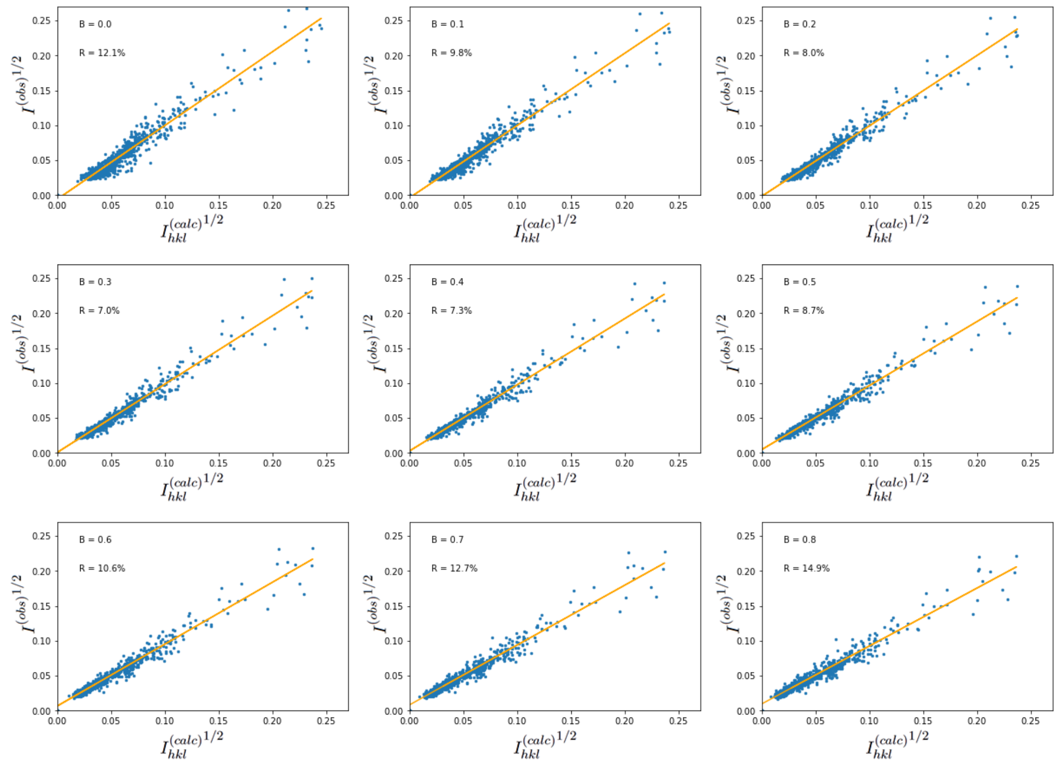

Fig. S7. R_1 calculations as a function of B for an optimised dynamical simulation as shown in Fig. 11. The lowest R is found at $B = 0.33$.

S1.7. Simulated best thickness plot

There is a lot of scatter in the plot, presumably because there are only a few reflections in each simulation, but there is clearly a trend that matches the expected plot.

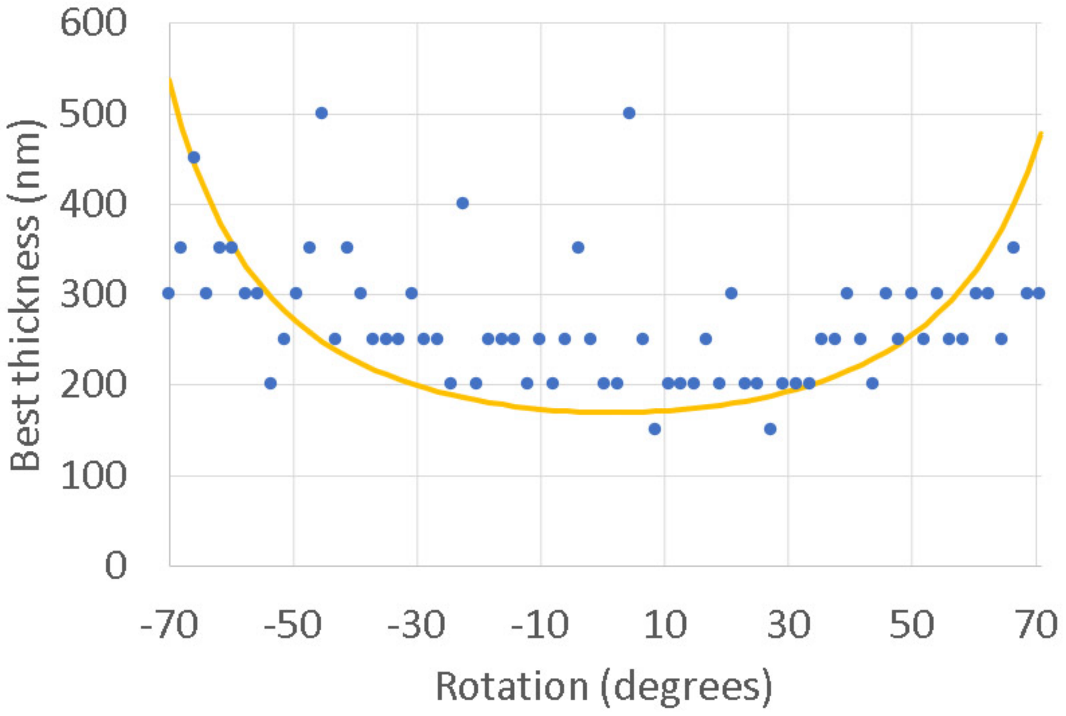

Fig. S8. The plot shows the best thickness (lowest R1) for each simulation together with the expected $1/\cos(\alpha)$ trend (orange line).
